# Eating at Food Outlets and “On the Go” Is Associated with Less Healthy Food Choices in Adults: Cross-Sectional Data from the UK National Diet and Nutrition Survey Rolling Programme (2008–2014)

**DOI:** 10.3390/nu9121315

**Published:** 2017-12-02

**Authors:** Nida Ziauddeen, Eva Almiron-Roig, Tarra L. Penney, Sonja Nicholson, Sara F. L. Kirk, Polly Page

**Affiliations:** 1 MRC Elsie Widdowson Laboratory, 120 Fulbourn Road, Cambridge CB1 9NL, UK; ealmiron@unav.es (E.A.-R.); Sonja.Nicholson@mrc-ewl.cam.ac.uk (S.N.); Polly.Page@mrc-ewl.cam.ac.uk (P.P.); 2 Academic Unit of Primary Care and Population Sciences, Faculty of Medicine, University of Southampton, Southampton SO16 6YD, UK; 3 Centre for Nutrition Research, University of Navarra, Pamplona 31008, Spain; 4 UKCRC Centre for Diet and Activity Reseacrh (CEDAR), MRC Epidemiology Unit, University of Cambridge School of Clinical Medicine, Box 285 Institute of Metabolic Science, Cambridge Biomedical Campus, Cambridge CB2 0QQ, UK; Tarra.Penney@mrc-epid.cam.ac.uk; 5 Healthy Populations Institute, Dalhousie University, P. O. Box 15000, Halifax, NS B3H4R2, Canada; sara.kirk@dal.ca

**Keywords:** eating location, core food, non-core food

## Abstract

Eating location has been linked with variations in diet quality including the consumption of low-nutrient energy-dense food, which is a recognised risk factor for obesity. Cross-sectional data from 4736 adults aged 19 years and over from Years 1–6 of the UK National Diet and Nutrition Survey (NDNS) Rolling Programme (RP) (2008–2014) were used to explore food consumption patterns by eating location. Eating location was categorized as home, work, leisure places, food outlets and “on the go”. Foods were classified into two groups: core (included in the principal food groups and considered important/acceptable within a healthy diet) and non-core (all other foods). Out of 97,748 eating occasions reported, the most common was home (67–90% of eating occasions). Leisure places, food outlets and “on the go” combined contributed more energy from non-core (30%) than from core food (18%). Analyses of modulating factors revealed that sex, income, frequency of eating out and frequency of drinking were significant factors affecting consumption patterns (*p* < 0.01). Our study provides evidence that eating patterns, behaviours and resulting diet quality vary by location. Public health interventions should focus on availability and access to healthy foods, promotion of healthy food choices and behaviours across multiple locations, environments and contexts for food consumption.

## 1. Introduction

Diet related diseases are a leading cause of disability and premature death [1]. Although the prevention of these chronic diseases can be attained through improved diet—including reduced burden of cardiovascular disease [2,3], diabetes [4], certain types of cancer [5] and conditions such as overweight and obesity [6,7]—current global trends suggest a continued increase in the consumption of foods that are incompatible with current diet recommendation [8]. 

To support healthier diet intakes for the population, there is a need to shift the focus from individual determinants toward the policies and environmental forces that distribute risk across the population [9,10,11]. Specifically, characteristics of the local food environment including exposure to food outlets and frequency of foods consumed outside of the home has been associated with less healthy diets and increased body weight in adults [12,13,14,15]. 

With the combined increase in prevalence of access to out-of-home food outlets [16] and a time constrained population [17], out-of-home food consumption is on the rise [18] with approximately 20–25% of adults and children in the UK eating meals prepared out-of-home [19]. Frequency of out-of-home eating has been linked with higher body weight as well as diets high in fat and energy and low in micronutrient content [14,15,20,21,22]; a diet pattern described as containing increased empty calories through overconsumption of solid fats and added sugars [23], which has been hypothesized to displace important micronutrient intakes [24]. 

Out-of-home food sources are varied, and can include vending machines, takeaways, cafes, restaurants, pubs/bars, convenience stores [16], the workplace and foods consumed during commuting or travelling [25]. While the role of takeaway outlets on diet quality and weight status have been explored considerably [26,27,28], other out-of-home eating locations have been investigated to a lesser extent. A recent study exploring out-of-home eating among US adults showed an association between higher frequency of out-of-home eating with body mass index (BMI) and a negative association with fruit and vegetable consumption [29], while other work demonstrated an additional positive association with biomarkers for chronic disease [30]. In addition, a study examining the influence of eating location on nutrient intakes in Irish adults found that energy, protein, fat and carbohydrates were significantly greater at home than at work or out-of-home, however the contribution of fat to both total and food energy was greatest from takeaways [21]. Adding to this, a systematic review examined eating out-of-home and its association with dietary intake and found that out-of-home foods were a significant source of energy intake, specifically a higher total daily energy intake, energy contribution from fat and lower micronutrient intake [14]. Another systematic review of prospective studies exploring the association between out-of-home eating and anthropometric changes found that eating at fast-food outlets was associated with greater increase in body weight and waist circumference over time than eating at restaurants [31].

In combination, this evidence suggests a possible association between specific eating locations such as fast-food and full service restaurants, diet quality and weight status [31,32,33]. However, it is unclear whether frequency of consuming foods at other locations may contribute to increased weight status and poorer diet patterns, leaving a need to systematically explore the range of possible eating locations. Previous research using the US National Health and Nutrition Examination Survey (NHANES) data suggests that the home environment in particular may be a frequent location of consumption of empty calories bought elsewhere [34]. Research in the UK [19,28] and Europe [6,25] has mainly focused on out-of-home eating and did not include diet quality. However, both eating out and patterns of food consumption have been linked with income with a healthy diet being identified as more expensive and could thus contribute to this relationship.

Our aim was therefore to examine and characterise the food environment of UK adults, both at home and out-of-home, and the types of food consumed, for which the following questions were addressed: (1) What are the most common eating locations in adults? (2) What types of foods are consumed in each location and how much do they contribute to daily energy intakes? (3) How do these patterns fluctuate with age and other modulatory factors? In particular, whether income, individual traits, and drinking/smoking habits were significant predictors of eating location intake patterns was studied. We present a descriptive analysis of cross-sectional dietary data across a wide range of eating locations in a representative sample of adults in the UK aged 19 years and over alongside an investigation of potential modulatory factors. 

## 2. Materials and Methods

### 2.1. Sample

Data collected between 2008 and 2014 as part of the UK National Diet and Nutrition Survey (NDNS) Rolling Programme (RP) (Years 1–6) were analysed [35]. The NDNS is designed as a nationally representative sample to assess the diet, nutrient intake and nutritional status of the general population aged 1.5 years and above living in private households in the UK. The survey recruits a core sample of 1000 participants per year (500 children and 500 adults, aged ≥19 years). During this period, the sample was boosted in Scotland, Wales and Northern Ireland up to 600 participants per year (combined). The NDNS survey design and sampling methods (published elsewhere [36]) entail a random sample drawn from the Postcode Address File (a list of all addresses in the UK) from which addresses were clustered into primary sampling units (PSUs), small geographical areas based on postcode sectors, randomly selected from across the UK. From each PSU, 27 addresses were randomly selected and sent an introductory letter about the survey, following which interviewers then called to arrange a face-to-face visit to recruit participants (one adult and/or child per household). An average response rate of 54% was achieved. Data for all adults aged ≥19 years were included in this analysis.

### 2.2. Dietary Data

Dietary assessment was carried out using four-day estimated food diaries; all participants who completed three or more diary days were included (2%, 90 participants completed three diary days). Participants were asked to keep a record of everything eaten and drunk over four consecutive days, both at home and away from home, and to provide brand names, recipes for home cooked foods and information on portion sizes. Portion sizes were generally estimated and recorded in household measures (for example, glasses, cups, and spoonfuls); quantification was informed using standardised pictures provided in the diary, food labelling information, or proportions of recipes when provided. Packaging of any branded items consumed were requested to be returned with the diary to enable use of the weights and nutrition information on the label by the researchers during data coding.

The NDNS protocol requires three visits with each participant undertaken by trained interviewers: the first to place the diary and to administer questions via Computer Assisted Personal Interview (CAPI), the second a brief visit (or telephone contact) to provide support and check compliance during diary completion, and the third final visit to allow for diary review, editing for possible clarification or omissions prior to collection. 

NDNS food and drink diaries are coded by trained staff and processed using DINO (Diet in Nutrients Out) [37]. Each recorded item is assigned a suitable food and portion code in accordance with food composition data from the Department of Health’s (DH) NDNS Nutrient Databank. Where standard portion sizes are recorded in the diary using the pictures provided, portion sizes are assigned from the Food Standard Agency’s (FSA) portion size book [38]. For composite items which can be split into their component parts, for example sandwiches, each individual component is coded. A similar approach is applied to homemade dishes for which recipes had been provided in the diary and these were then linked together to indicate being cooked together.

For validation of estimations of energy intake from the self-reported dietary records of food and drinks consumed, the NDNS RP included a doubly labelled water (DLW) sub-study of participants aged four years and over [36]. 

### 2.3. Other Variables

BMI was calculated using height and weight measurements taken by the interviewer. Data on ethnicity, income, frequency of takeaway meal consumption, frequency of eating out, frequency of drinking and smoking were collected through self-report using questions that were designed specifically for the NDNS RP CAPI and carried out during the first diary placement visit.

For Frequency of takeaway meal consumption and eating out, the questions were “On average, how often do you/does child eat take-away meals at home?” and “On average, how often do you/does child eat meals out in a restaurant or cafe?”. In both questions, the interviewer specified that “‘meals’ means more than a beverage or bag of chips” and participants were asked to “include pizza, fish and chips, Indian, Chinese, burgers, kebab etc.”. The following response options were available to the participant: “rarely or never”, “1–2 times per month”, “1–2 times per week”, “3–4 times per week”, and “5 or more times per week”. 

For drinking and smoking, participants aged 18–24 years were given the option of filling out a self-completion booklet or answering the interviewers CAPI questions, both of which included 11 smoking and 42 drinking questions to determine units, nature and frequency of drinking alcohol. 

### 2.4. Defining Eating Occasions

In addition to details of what and how much was eaten, for each eating occasion participants, were asked to record: time of eating, where they were, who they were eating with, whether they were watching TV, and/or sitting at a table. Individual food data by time slot were analysed to capture the eating location. All occasions consumed within 15 minutes of each other were combined into a single eating event [39] provided the eating location remained the same. For the purpose of this study, detailed eating location data were aggregated into five broad categories as follows:

- Home: bedroom, dining room, garden, kitchen, living room, home/other, home/unspecified
- Work: all work canteen categories, desk, work/other. Eating occasions at university (all university canteen categories, university/other) were combined with work due to the small number of eating occasions in this location (0.2% of all occasions) which did not allow for meaningful interpretation
- Leisure places: sports clubs, sports leisure venue, leisure activity place /cinema/shopping centre/place of interest, attractions, community/day centre, public hall, function room
- Food outlets: restaurant/pub/night club, fast food outlet, coffee shop/cafe/deli/sandwich bar
- “On the go”: bus, car or train outside/other, street

Eating occasions at other locations (3.7% of all occasions) such as friend’s and relatives’ house, holiday accommodation, other place and place of worship have been excluded from this analysis as this represented a non-homogenous mixture of locations. Unspecified locations included home-unspecified (categorised as home), or unspecified (excluded, 1.1% of all occasions). The number of entries in each of these locations was not sufficient to split into separate analyses and hence does not allow for meaningful interpretation as a separate location group. 

### 2.5. Dietary Variables and Classification of Foods as Core or Non-Core

Each food consumed in NDNS RP was defined as a core or non-core food based on a previously published classification [40] (Table 1). Core foods were defined as those included in the principal food groups and considered important or acceptable within a healthy diet such as cereals and cereal products, meat (excluding processed meat), meat alternatives, fish, vegetables, fruit, nuts/seeds and dairy products. All other foods were classified as non-core foods and included pastries, cakes, high-fat snacks and sugary drinks amongst other foods [40]. 

We also selected a range of foods and nutrients of public health interest for the UK population for specific examination. Fruit and vegetables and fibre are consumed in insufficient amounts whereas it is recommended that the intakes of red and processed meat, sugar sweetened beverages (SSB), non-milk extrinsic sugars (NMES) and saturated fatty acids should reduce [41,42]. Fruit and vegetable consumption figures have been calculated using disaggregated data [43] and the number of portions have been calculated in line with the “5-a-day” guidelines of 80 g per portion (including one 150 mL portion of fruit juice) [44]. As the Englyst method for the determination of amount of fibre in food was in use in the UK during the period of data collection, this is the fibre value that has been used for this analysis. SSB include concentrated, still and carbonated soft drinks with added sugar. 

### 2.6. Data Analysis

NDNS RP data are weighted to adjust for differences in sample selection and response. All analyses were carried out using the survey package in R v3.0.2 (Vienna, Austria) [45] to account for the stratification and clustering in the NDNS RP sample design.

A descriptive analysis of percentage of eating locations, core and non-core food consumption and energy intake was carried out. Mean intakes over the four days (or three days in those who only filled out three days) in grams of fruit and vegetables, red and processed meat, SSB and fibre and as a percentage of total energy of NMES and saturated fatty acids were calculated at each location. NMES and saturated fatty acid intakes are presented as a percentage of total energy intake so that comparisons to recommended daily energy intakes can be made. Due to an unequal distribution of eating occasions per location, the intakes of selected foods and nutrients in each location as a proportion of overall food/drink (foods) and energy intake (nutrients) respectively in that location was calculated for each participant and presented as a population mean. Energy intakes from core and non-core foods by eating location were compared using logistic regression using home as the reference location. Intakes of selected foods and nutrients within an age group were also compared using logistic regression for the likelihood to consume more fruit and vegetables, red and processed meat, SSB, fibre, NMES and saturated fatty acids using home as the reference location. For both analyses, patterns between age groups were compared for consistency (the interaction between age group and pattern of intake was tested).

To investigate the impact of modulatory factors on eating location patterns, the sample was then split into quintiles by the percentage consumption of meals at home, that is those consuming <25%, 25–49%, 50–69%, 70–89% and ≥90% of meals at home. This approach was taken after initial exploration of the data which indicated that home was the most frequent eating location, and although meals were also consumed in other locations the small number of occasions did not allow for the characterisation of these individual locations. We also considered that adults consuming ≥90% of meals at home are likely to differ from those consuming fewer meals at home. The intakes for saturated fatty acids, NMES, fibre and fruit and vegetables were compared against recommendations by home meal pattern category within a number of age groups. We also examined the impact of potential confounders such as sex, ethnicity, BMI, income, frequency of takeaway meal consumption, frequency of eating out, frequency of drinking and smoking on home meal pattern categories using multiple regression analysis. Interactions amongst these factors were explored when appropriate. The level of statistical significance for all analysis was set at *p* < 0.05.

## 3. Results

**Figure 1:**
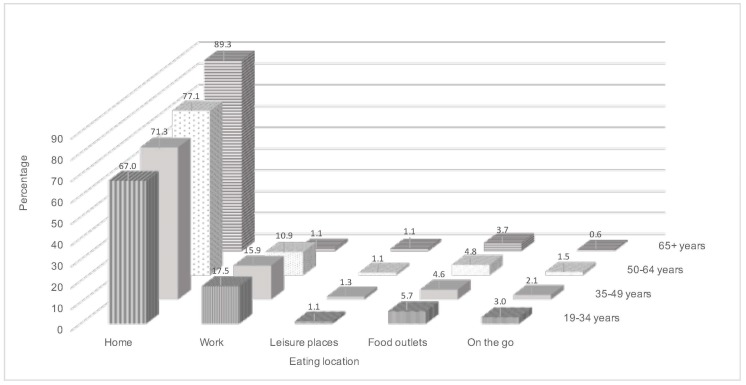
Percentage of reported eating location by age group for the National Diet and Nutrition Survey (NDNS) Rolling Programme (RP) Years 1–6 (2008–2014) adult population.

The sample consisted of 4736 adults aged 19–96 years with a total of 97,748 reported eating occasions. Results are presented by age groups (1067 aged 19–34 years, 1421 aged 35–49 years, 1174 aged 50–64 years and 1074 aged ≥65 years). The most frequent eating location across all age groups was home (67.0–89.3% of eating occasions) with the percentage of occasions at home increasing with age (Figure 1). After home, except for those aged 65 and over, work was the second highest location category for eating occasions in all age groups, decreasing from 17.5% and 15.9% in the two younger age groups (19–34 and 35–49 years respectively) to 10.9% in adults aged 50–64 years. Work represented 1.1% of occasions in those aged 65 years and over. The percentage of eating occasions at locations outside of home and work were much lower in comparison and decreased with age for all locations except leisure places which remained comparable across all age groups.

**Figure 2:**
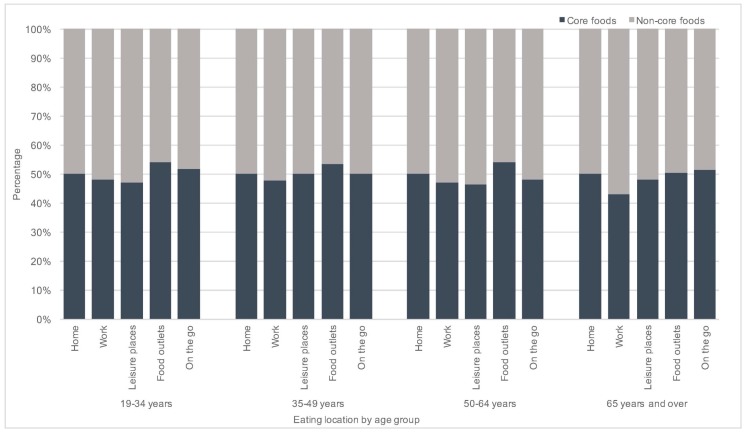
Percentage consumption of core and non-core foods by reported eating location for the NDNS RP Years 1–6 (2008–2014) adult population.

The consumption of core and non-core foods at home was similar across age groups (Figure 2). The pattern at work was similar, with slightly lower core food than non-core food consumption for all age groups. Leisure places contributed slightly more to non-core food than core food consumption across all age groups except adults aged 35–49 years where the contribution was equal for core and non-core foods. The opposite was observed in food outlets where core food consumption was slightly higher than non-core food in all age groups excepts those aged 65 years and over. The consumption of core and non-core foods “on the go” was similar for all age groups.

**Figure 3:**
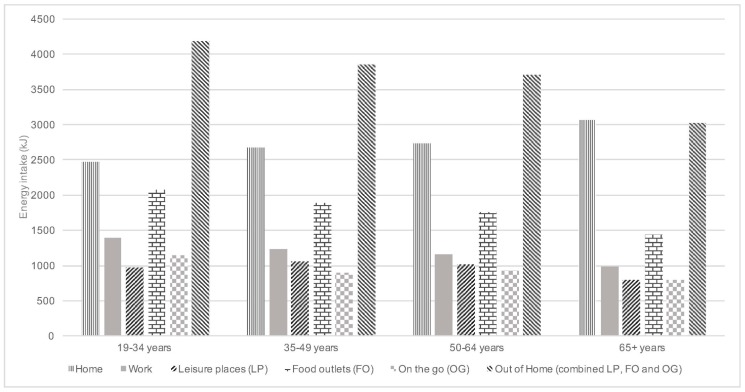
Energy intake (kJ) by reported eating location for the NDNS RP Years 1–6 (2008–2014) adult population.

**Figure 4:**
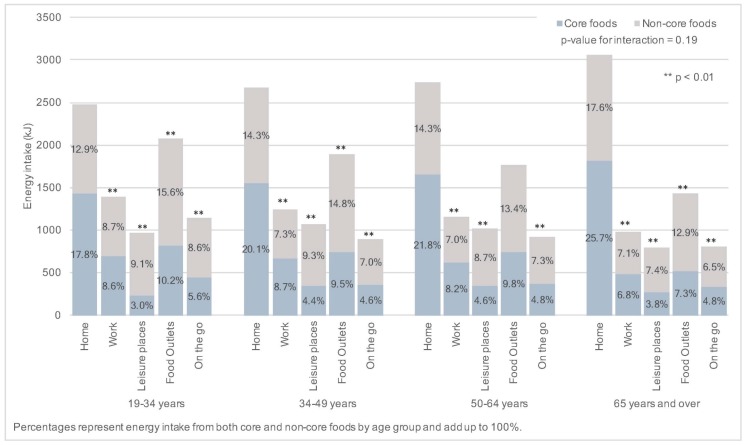
Energy intake (kJ) from core and non-core foods by reported eating location for the NDNS RP Years 1–6 (2008–2014) adult population. Results of logistic regression comparing energy intakes from core and non-core foods by eating location within age group using home as the reference location are presented using asterisks.

Across all age groups, the two locations with the highest energy intake (Figure 3) and highest non-core food energy intake were food outlets and home. When combined, leisure places, food outlets and “on the go” contributed the highest amounts of energy in all age groups up to age 65, when they contributed equal amounts as home. Non-core food energy intake across locations decreased with age from 55% of total energy intake in adults aged 19–34 years to 52% in adults aged 65 years and over (Figure 4). The contribution of core food energy intake at home to overall energy intake increased with age from 17.8% in adults aged 19–34 years to 25.7% in adults aged 65 years and over and the difference in patterns across age groups was significant (*p* < 0.01). Leisure places, food outlets and “on the go” combined contributed more to non-core than core food energy intake across all age groups (30.2% compared to 18.1%). Work contributed a similar amount to core and non-core food energy intake in adults aged 19–34 years and slightly less non-core food energy in adults 35–64 years with non-core food energy intake decreasing with age from 8.7% in adults aged 19–34 years to 7.1% in adults aged 65 years and over, however only 1.1% of all eating occasions was at work in this age group compared to 10.9–17.5% in other age groups. With the exception of food outlets in adults aged 50–64 years, intake across each location was significantly different from home across all age groups (*p* < 0.01). 

### 3.1. Intakes of Selected Foods and Nutrients by Eating Location

Average intakes of selected foods and nutrients as a percentage of overall consumption and energy intake respectively by location are presented in Table 2. 

*Fruit and vegetables*: The highest proportion of fruit and vegetables was consumed “on the go” across all age groups, followed by home and work. However, consumption “on the go” was only significantly higher than home in adults aged 35–49 and 50–64 years. As a proportion of overall consumption, fruit and vegetable consumption at home increased with age. A similar trend was seen at work across all age groups up to 64 years following which it decreased, reflective of the lower number of eating occasions at this location due to retirement. Consumption at leisure places and food outlets was significantly lower than at home in all age groups. 

*Red and processed meat*: The highest proportion of red and processed meat was consumed in food outlets across all age groups significantly so in adults 35–49 and 65 years and over. The lowest proportion was consumed at work across all age groups (except in those aged 50–64 years) and remained significant compared to consumption at home across all age groups. 

*Sugar sweetened beverages (SSB)*: The highest proportion of SSB was consumed by adults aged 19–34 years across all locations and consumption was significantly higher in all locations compared to home in this age group. Leisure places, food outlets and “on the go” were the locations with the highest proportion of consumption of SSB across all age groups. The proportion of consumption of SSB decreased in adults aged 35 years and older compared to younger adults. The proportion of consumption by age group was significantly different across the locations (*p* < 0.01).

*Fibre*: Fibre intake, as a proportion of overall intake by location, was highest “on the go” across all age groups with home being the location with the next highest intake. Consumption “on the go” was significantly higher than at home across all age groups. 

*Non-milk extrinsic sugars (NMES):* NMES intake as a percentage of total energy across locations decreased with age from those aged 19–34 years to those aged 50–64 years but then increased slightly in adults aged 65 years and over. However, the difference in the proportion of consumption across locations was not significant across age groups (*p* = 0.97). Intakes were comparable across locations in adults aged 19–34 years and 35–49 years. NMES intakes at work were lowest in adults aged 50 years and over. NMES intakes at home in adults aged 35 years and over was the only location where the recommendation for NMES intake of less than 11% of total energy [46] was met.

*Saturated fatty acids*: Intake of saturated fatty acids as a percentage of total energy was significantly lower “on the go” than at home across all age groups. Overall, saturated fatty acid intake remained high and in excess of the recommendation of no more than 11% total energy [46] at home and work in adults aged 19–64 years and in most locations in adults aged 50 years and over. The proportion of consumption by age group was significantly different across the locations (*p* < 0.01).

### 3.2. Home Eating Consumption Patterns and Impact of Modulatory Factors

**Figure 5:**
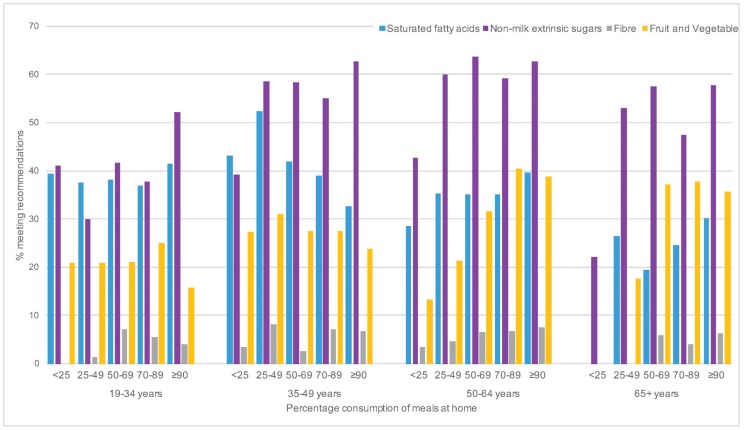
Percentage of adults meeting the recommended intakes of saturated fatty acids, non-milk extrinsic sugars and fibre split by home meal consumption pattern for the NDNS RP Years 1–6 (2008–2014) adult population.

The comparison of intakes against recommendations by categories of percentage consumption of meals at home for saturated fatty acids, NMES and fibre is presented in Figure 5 In general, the percentage meeting NMES recommendations increased with increasing percentage of meals at home across all age groups though there were fluctuations within each category. The percentage of adults meeting “5-a-day” recommendations for fruit and vegetables also increased with increasing percentage of meals eaten at home but only in those aged 50 years and over. No clear pattern was seen with fibre intake except in adults aged 50–64 years where the percentage meeting recommendations increased with increasing percentage of meals consumed at home mirroring fruit and vegetable consumption. However the overall percentage of adults meeting the recommendation for fibre remained low. No clear pattern was observed for meeting recommendations of saturated fatty acids intake and percentage of meals consumed at home.

Regression analyses of modulating factors revealed that sex, income, frequency of eating out and frequency of drinking were significant factors affecting consumption patterns (Table 3) (model included sex, ethnicity, income, frequency of eating out, frequency of takeaway meal consumption, BMI, frequency of drinking alcohol and smoking). Women were more likely than men to consume ≥70% of meals at home (*p* = 0.01). Income was a significant factor affecting consumption patterns with those in the lower income quintiles more likely to have a higher percentage of meals consumed at home. In terms of eating out, the highest proportion of participants ate out 1–2 times per month across all meal consumption groups but the proportions decreased across the groups from those who consumed 25–49% meals at home. Less frequent takeaway meal consumption (1–2 times per week and per month) significantly affected consumption patterns with proportions of those lowest in those who consumed ≥70% of meals at home in these two takeaway meal categories. Drinking once a week or more was a significant factor with a high proportion less likely to consume a high proportion of meals at home. 

## 4. Discussion

This analysis shows that most of the energy intake for this sample of adults living in the UK came from foods eaten at home and from a variety of foods including both healthy and less healthy options. As adults aged, they ate more frequently at home thus having a similar percentage consumption of core and non-core foods but with core foods contributing a higher percentage to energy intake. While the percentage consumption of core vs. non-core food was not very different by location, the difference in energy provided by each of these two groups of foods was however important. In particular, while at home the contribution of non-core food energy was always lower than that for core food energy, the reverse occurred for the combined food outlets, leisure places and "on the go" context. Additionally, energy intake was disproportionately higher at leisure places, food outlets and “on the go” combined across all age groups. Foods frequently consumed in these locations included nutrient-poor energy dense choices such as spreads, cakes, savoury snacks and SSB (non-core foods), and other sources of saturated fatty acids and sugar, confirming results from other countries [14,20,25]. Approximately a quarter of daily total energy intake came from non-core foods from these locations across all age groups with core foods only contributing around 18% in the same locations. These trends reflect the potential impact that specific food environments (such as those lacking affordable healthy food choices with an abundance of cheap, non-core food) can have on food choices, which could in turn undermine health-promoting government messages.

Fruit and vegetable consumption was highest “on the go” across all age groups which is likely to be reflective of the small proportion of eating occasions in this location compared to other locations, and thus a smaller variety but larger amount of foods is likely to be consumed in this location. This could also reflect a higher proportion of fruit eaten “on the go” and probably juice consumed at leisure places, although such foods were also consumed in similar proportions in other locations. Fibre followed a similar pattern but overall intakes were low. Indeed, mean intake of fruit and vegetables and fibre for the whole NDNS RP adult population are below recommendations [36] and while our analysis does not allow to pinpoint specifically where intake was lowest, it is possible that fruit and vegetable intake is displaced by higher intakes of processed foods and SSBs, especially out of the home [28,34]. A high consumption of NMES across all out-of-home locations and age groups, red and processed meats in leisure places, food outlets and “on the go”, and SSBs in leisure places and “on the go” was observed which would support this assumption. The same was observed for saturated fatty acids intake which was in excess of the recommendations [46] across all age groups and locations. 

While there was no clear association between consumption of saturated fatty acids and fibre and how many meals were eaten at home, those consuming more meals at home were less likely to exceed recommendations for NMES, and in adults aged 50 years and over more likely to meet 5-a-day recommendations. This suggests that reduced exposure to the out-of-home food environment may positively influence some food choices, as previously proposed [28,34,47]. 

This analysis used a nationally representative sample of adults in the UK including >95,000 eating occasions and explored a wide range of eating locations as well as food and nutrient sources, providing sound evidence for a link between eating location and consumption. A limitation of our study is that the core and non-core food classification may not be specific enough, for example the inclusion of fruit juice, sweetened dairy products and refined grains as core foods [42]. However, sweetened dairy products contributed a small proportion to total intake of dairy products and the impact of their inclusion within core-foods is likely to be small. Fruit juice intake within the classification was capped at the recommended portion of 150 mL per day in accordance with the UK government “5-a-day” guideline. While refined grains contribute less fibre and nutrients to the diet than whole grains and tend to over-dominate dietary intakes, their inclusion as a core food reflects the major contribution of these foods to the diet [48]. Another limitation was the inability to control for factors such as physical activity, dieting practices or illness that may affect food choices. Finally, as with all self-reported dietary data, a degree of reactivity to recording food consumption [49] and other dietary misreporting cannot be excluded in this population. In the NDNS RP DLW sub-study, the agreement between energy intake and total energy expenditure was 0.68 for participants aged 16 years and over. 

The patterns of core and non-core food intake in adults are not strikingly different between the age groups in contrast to differences observed across the age groups in children [50]. However, habits once formed in childhood are generally tracked through adulthood [51]. As childhood obesity rates have been on the rise [52], it is plausible that eating habits in children now may be worse compared to when the adults in the survey were children. Our findings support a number of previous studies including a previous NDNS RP analysis suggesting that the home environment can be a location for both healthy and less healthy food, especially takeaway food, including pizza, SSB and alcohol purchased at food outlets and supermarkets and brought into the home [19,34]. An important difference we found was that consumption of SSBs at home was markedly lower than comparable data from the USA [34]; however, that study was based on purchase data as a proxy for consumption, while our study is based on actual reported intake. 

The mechanisms by which consumption of less healthy foods may be linked with specific locations such as fast food and takeaway outlets are not completely known, but the particular eating location itself may favour the clustering of specific food behaviours [53]. This may be due to the range of foods offered which may be limited due to perishability, closeness to work places, taste preferences, and cost of food [54,55]. The association between low income and poorer diet quality has been extensively reported and our findings corroborate these studies [56,57]. Beyond the effect of the food environment per se, we found evidence that those consuming more meals at home generally tended to be older, of a lower income, consume takeaway food no more than twice a week, and drink alcohol no more than once a week. 

## 5. Conclusions

Although energy intake from core foods were higher at home across all age groups, eating out-of-home, particularly in food outlets, leisure places and “on the go”, was linked with higher energy intakes from foods associated with a dietary pattern that is incompatible with current guidelines. Changes in number of eating occasions, energy density and portion size have been shown to be key contributors to changes in total energy intake in USA and thus further investigation into these factors in the UK could be of interest [39]. Our study did not investigate which specific mechanisms trigger these associations and future research on the food source and determinants of food choice across eating location is necessary. Our results however support public health interventions to increase availability and access to healthy food in leisure places, workplaces and food outlets, especially those providing food to eat “on the go”. Our parallel analysis in children confirms these findings [50].

## Figures and Tables

**Table 1:** National Diet and Nutrition Survey (NDNS) Rolling Programme (RP) food groups defined as core and non-core.

Core foods	Non-core foods
Pasta, rice and other miscellaneous cereals	Biscuits
Bread (all types)	Buns, cakes, pastries and fruit pies
Breakfast cereals (all types)	Puddings
Milk (all types)	Ice cream
Cheese	Butter, spreads and oil
Yogurt and fromage frais	Dairy desserts
Eggs and egg dishes	
Beef, veal and dishes	Meat pies and pastries
Lamb and dishes	
Pork and dishes	Bacon and ham
Chicken and turkey dishes	Coated chicken and turkey
Liver products and dishes	
	Burgers and kebabs
	Sausages
	Other meat and meat products
White fish, shellfish and fish dishes	Coated or fried white fish
Oily fish	
Salad and other raw vegetables	
Vegetables (not raw) including beans and meat alternatives	
Other potatoes and potato salads	Chips, fried and roast potatoes and potato products
Nuts and seeds	
Fruit	
Smoothies	
Fruit juice (capped at maximum intake contribution to 5-a-day)	Soft drinks not diet
Tea, coffee and water	Soft drinks diet
	Alcoholic beverages
	Sugar, preserves and sweet spreads
	Sugar confectionery
	Chocolate confectionery

**Table 2:** Consumption of selected foods and nutrients by reported eating location from the NDNS RP Years 1–4 (2008–2012), as a percentage of overall consumption per location and age group. Results of logistic regression are indicated under the respective *p* columns using Home as the reference category indicated as “(ref)”. The interaction between age group and selected foods and nutrients was tested using the same models and is shown at the bottom of the table.

		Fruit and Vegetables		Red and Processed Meat		Sugar Sweetened Beverages		Fibre		Non-Milk Extrinsic Sugars (NMES)		Saturated Fatty Acids	
		Mean % (Food Intake)	*p*	Mean % (Food Intake)	*p*	Mean % (Food Intake)	*p*	Mean % (Food Intake)	*p*	Mean % (Energy Intake)	*p*	Mean % (Energy Intake)	*p*
19–34 years	Home	10.4	(ref)	3.1	(ref)	12.6	(ref)	0.6	(ref)	12.0	(ref)	12.3	(ref)
	Work	9.5	0.16	1.7	0.00	14.3	0.00	0.5	0.00	16.4	0.00	11.7	0.18
	Leisure places	4.2	0.00	2.1	0.12	13.9	0.00	0.5	0.27	23.8	0.00	8.4	0.00
	Food outlets	6.8	0.00	3.7	0.18	17.4	0.00	0.5	0.00	16.4	0.00	9.3	0.00
	On the go	12.3	0.16	2.7	0.38	21.2	0.00	0.7	0.00	23.3	0.00	11.1	0.04
35–49 years	Home	11.0	(ref)	2.9	(ref)	6.5	(ref)	0.6	(ref)	10.4	(ref)	12.1	(ref)
	Work	10.6	0.40	1.7	0.00	5.8	0.00	0.5	0.00	13.8	0.00	12.0	0.71
	Leisure places	4.1	0.00	2.2	0.11	7.4	0.00	0.5	0.36	19.4	0.00	9.7	0.00
	Food outlets	6.9	0.00	4.0	0.00	8.6	0.00	0.4	0.00	14.8	0.00	10.0	0.00
	On the go	15.3	0.00	2.3	0.13	11.0	0.00	0.8	0.00	20.2	0.00	9.7	0.00
50–64 years	Home	12.2	(ref)	2.7	(ref)	3.6	(ref)	0.6	(ref)	9.9	(ref)	12.4	(ref)
	Work	11.1	0.10	2.0	0.00	2.8	0.00	0.5	0.00	13.0	0.00	12.6	0.59
	Leisure places	6.6	0.00	2.2	0.44	6.4	0.00	0.4	0.05	19.1	0.00	11.5	0.28
	Food outlets	7.7	0.00	3.4	0.06	5.8	0.00	0.5	0.00	14.0	0.00	10.4	0.00
	On the go	18.6	0.00	1.7	0.00	7.7	0.00	0.9	0.00	19.7	0.00	10.4	0.01
≥65 years	Home	12.7	(ref)	2.6	(ref)	3.1	(ref)	0.6	(ref)	10.8	(ref)	13.2	(ref)
	Work	7.3	0.00	1.3	0.00	0.2	0.06	0.4	0.00	11.7	0.61	13.6	0.71
	Leisure places	6.9	0.00	2.6	0.99	6.7	0.00	0.3	0.00	20.4	0.00	13.8	0.57
	Food outlets	7.5	0.00	3.4	0.04	4.9	0.00	0.4	0.00	13.6	0.00	12.3	0.08
	On the go	13.3	0.83	2.6	0.95	3.7	0.01	0.9	0.00	23.4	0.00	10.3	0.00
*p* for trend		0.22		0.29		0.00		0.25		0.97		0.00	

**Table 3:** Characteristics of the NDNS RP Years 1–4 (2008–2012) adult population by consumption pattern of meals at home.

		Percentage Consumption of Meals at Home					*p* Value
		<25	25–49	50–69	70–89	>90	
*n*		88	477	1047	1587	1537	
Age (years) (mean)		37.9	37.2	41.0	49.4	56.5	
Sex (%)	Male	65.3	55.0	56.2	43.6	44.8	(ref)
	Female	34.7	45.0	43.8	56.4	55.2	0.01
Ethnicity (%)	White	94.8	91.7	90.1	89.4	87.8	(ref)
	Non-white	5.2	8.3	9.9	10.6	12.2	0.57
Income (quintile) (%)	≤£12,300	6.6	6.2	9.3	16.8	23.6	(ref)
	>£12,300 ≤ £19,890	6.3	13.0	16.4	19.9	28.8	0.04
	>£19,890 ≤ £28,615	22.0	17.6	19.3	18.7	16.3	0.00
	>£28,615 ≤ £42,500	18.6	30.2	23.3	22.0	17.5	0.00
	>£42,500	46.5	33.0	31.6	22.6	13.8	0.00
BMI (%)	Underweight	0.6	2.2	1.3	0.6	2.3	0.85
	Normal weight	39.7	38.8	34.2	35.4	33.8	(ref)
	Overweight	25.4	35.8	39.3	35.3	36.7	0.44
	Obese	30.5	19.5	22.9	26.0	25.0	0.66
	Morbidly obese	3.8	3.7	2.3	2.7	2.2	0.04
Frequency of eating out (%)	5 or more times per week	4.7	3.7	2.1	1.1	0.5	0.00
	3–4 times per week	8.2	5.3	5.3	2.8	2.4	0.00
	1–2 times per week	30.1	27.6	26.1	24.3	15.3	0.00
	1–2 times per month	38.3	47.9	45.6	44.9	39.2	0.00
	Rarely or never	18.7	15.5	21.0	26.9	42.6	(ref)
Frequency of takeaway meals (%)	5 or more times per week	0.8	0.5	0.1	0.2	0.6	0.47
	3–4 times per week	0.7	1.7	1.2	1.0	1.6	0.62
	1–2 times per week	23.9	30.5	22.8	16.9	14.1	0.00
	1–2 times per month	51.9	40.0	45.3	35.5	27.0	0.00
	Rarely or never	22.6	27.3	30.6	46.5	56.7	(ref)
Drinking (%)	Once a week or more	75.4	63.2	57.2	51.1	38.7	0.00
	Once or twice a month	11.0	16.9	17.3	17.6	16.0	0.34
	Once every couple of months	2.0	5.0	7.6	10.5	10.1	0.98
	Few times a year	5.2	5.4	6.9	8.4	12.7	0.60
	Never drinks	6.5	9.5	11.0	12.4	22.5	(ref)
Smoking (%)	No	71.4	74.6	72.9	78.8	76.1	(ref)
	Yes	28.6	25.4	27.1	21.2	23.9	0.06
